# Supplementary material for: Early cellular innate immune responses drive Zika viral persistence and tissue tropism in pigtail macaques
Source: Nat Commun. 2018 Aug 22;9:3371. doi: 10.1038/s41467-018-05826-w (PMC6105614; doi:10.1038/s41467-018-05826-w)
Supplement: Supplementary file 1 — Supplementary Information [file 41467_2018_5826_MOESM1_ESM.pdf]

Early cellular innate immune responses drive Zika viral persistence and tissue tropism in pigtail macaques

O'Connor et al

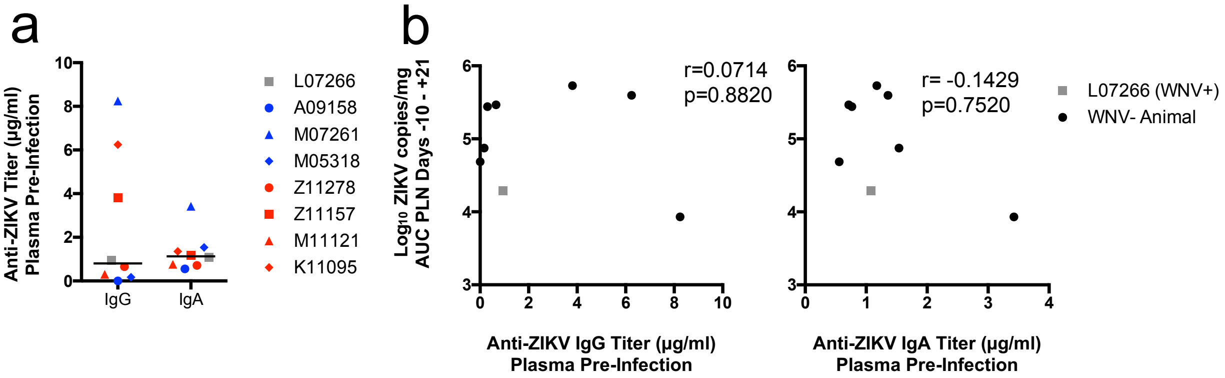

Supplementary Figure 1. Pre-infection anti-ZIKV IgG and IgA responses in plasma. (a) Pre-infection plasma IgG and IgA levels against the Zika E protein were quantified by ELISA and titers were calculated from a standard curve. Symbols represent individual animals and the line indicates the median. WNV^{-} females (blue), WNV^{+} female L07266 (gray), WNV^{-} males (red) (b) Pre-infection anti-ZIKV IgG (left panel) or IgA (right panel) against the E protein in one WNV^{+} female L07266 (gray square) and the 7 WNV^{-} male and female animals (black circles) do not correlate with viral burden in the PLN (AUC, days -10 to +21). Spearman's rank correlation coefficient and unadjusted p-value are shown (both are not significant).

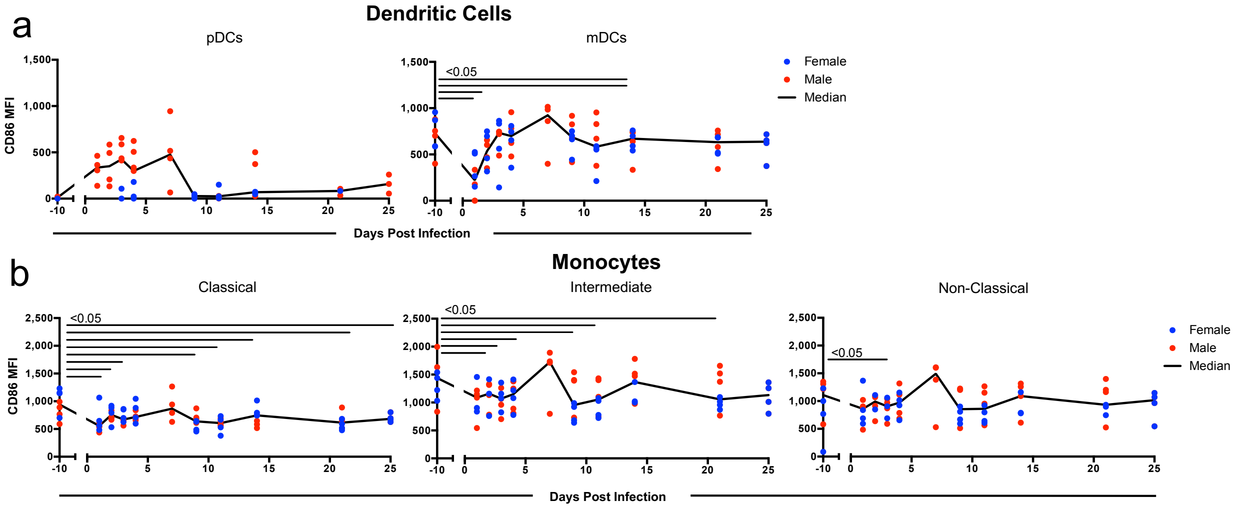

Supplementary Figure 2. Downregulation of CD86 on several innate immune cell types in the whole blood. In the whole blood, flow cytometry was used to measure the kinetics of CD86 expression (mean fluorescence intensity, MFI) on (a) dendritic cell and (b) monocyte cell subsets throughout ZIKV infection. Blue (females) and red (males) dots are individual animals. The line indicates the median of all animals. Comparisons of responses at each timepoint versus baseline were conducted by paired Wilcoxon test. Unadjusted p-values ≤0.05 are displayed, all p-values are available in **Supplementary Table 3**. Statistical analysis of CD86 MFI on pDCs was not possible (indicated as N/A in **Supplementary Table 3**), because the frequencies of cells in some animals at some timepoints were below the minimum threshold.

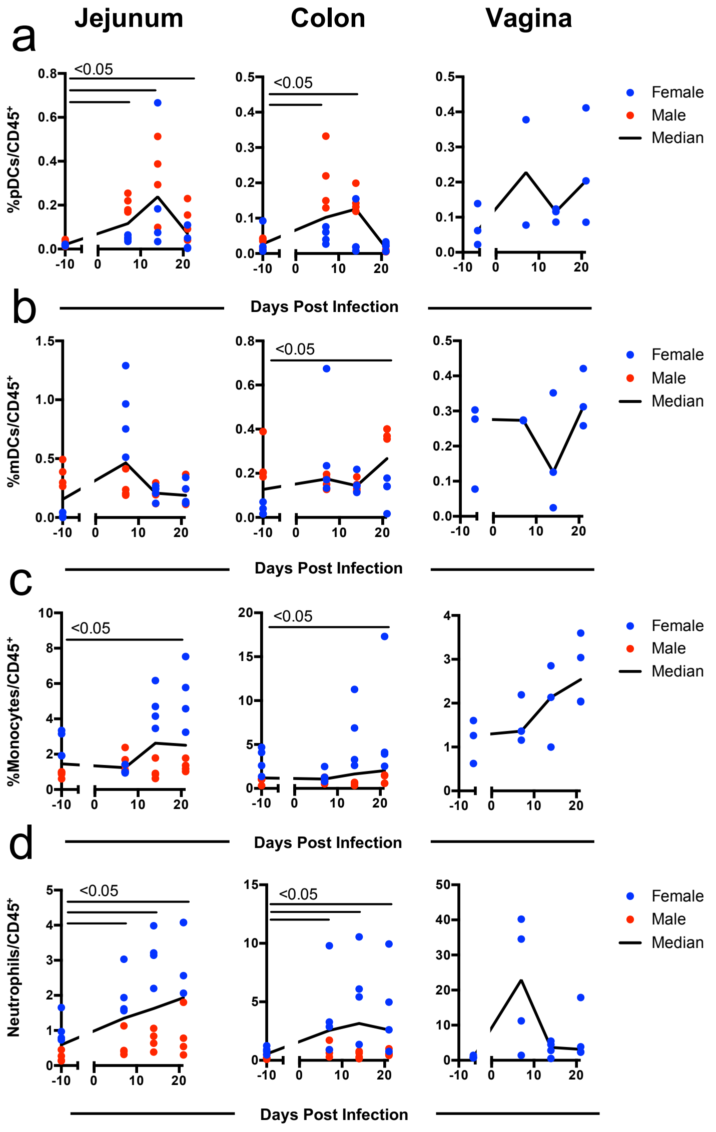

Supplementary Figure 3. Recruitment of innate immune cells to tissues lacking ZIKV infection. Frequencies of (a) pDCs (CD20^{-}CD3^{-}HLA-DR^{+}CD14^{-}CD123^{+}CD11c^{-}), (b) mDCs (CD20^{-}CD3^{-}HLA-DR^{+}CD14^{-}CD123^{-}CD11c^{+}), (c) monocytes (CD20^{-}CD3^{-}HLA-DR^{+}CD16$^{\pm}$CD14$^{\pm}$), and (d) neutrophils (CD3^{-}CD11b^{+}CD14^{+}HLA-DR^{-}SSC-A^{Hi}), within CD45^{+} leukocytes in the jejunum, colon, and vagina were measured by flow cytometry prior to ZIKV infection (day -10 for gut; day -2 for vagina) and 7, 14, and 21 dpi. Blue (females) and red (males) symbols represent individual animals, with curves indicating the median response for all

animals over time. Comparisons of responses at each timepoint versus baseline were conducted by paired Wilcoxon test. Unadjusted p-values ≤0.05 are displayed and all p-values are available in **Supplementary Table 2**. Statistical analysis in the vagina of certain subsets was not possible (indicated as N/A in **Supplementary Table 2**), because the frequencies of cells in some animals at some timepoints were below the minimum threshold.

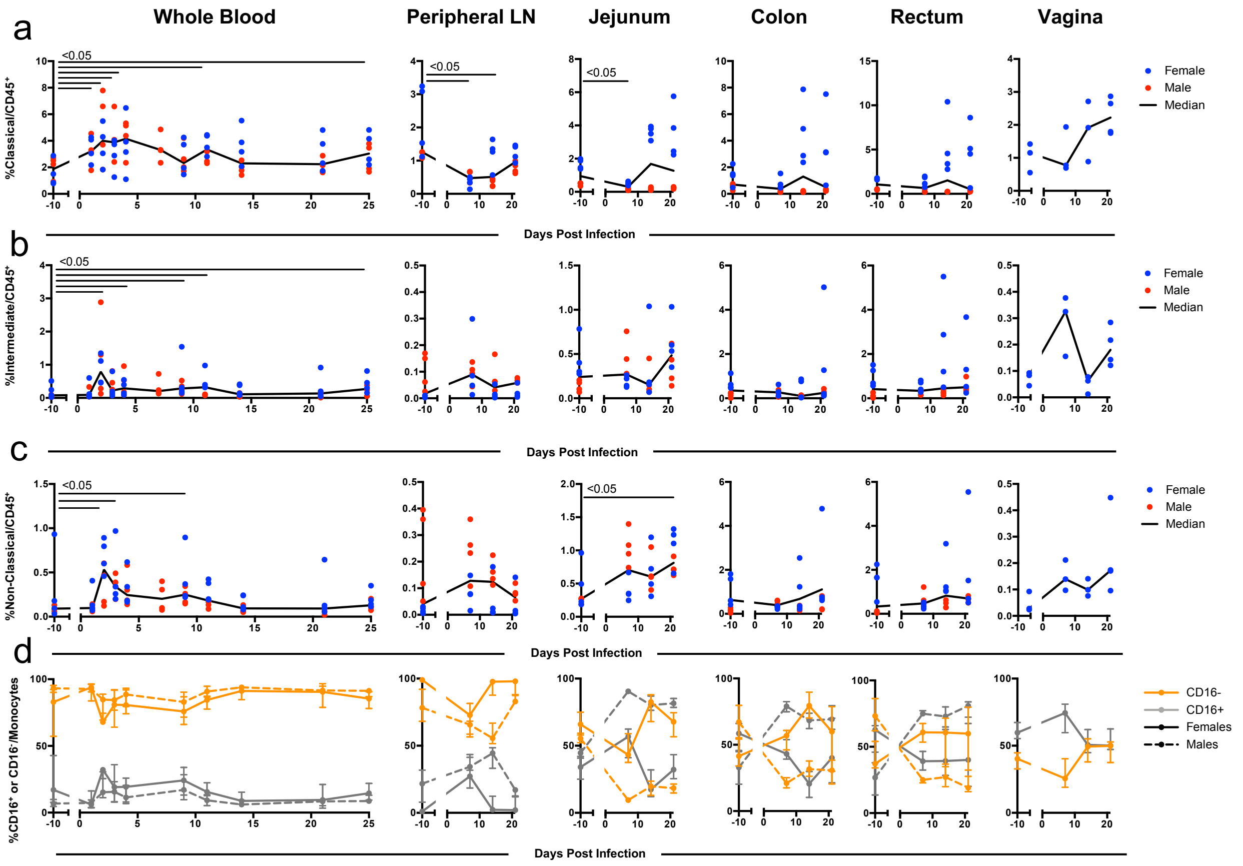

Supplementary Figure 4. Recruitment of monocyte subsets to blood and mucosal tissues. Monocyte subset frequencies within CD45$^+$ leukocytes before and following ZIKV infection in the whole blood, peripheral LN, and mucosal tissues (jejunum, colon, rectum, vagina) was determined by flow cytometry. Monocytes subsets were defined as: (a) classical (CD16$^-$CD14$^+$), (b) intermediate (CD16$^+$CD14$^+$), and (c) non-classical (CD16$^+$CD14^{int}). Blue (females) and red (males) symbols represent individual animals, with the curve indicating the median response of all animals over time. Comparisons of responses at each timepoint were conducted and all unadjusted p-values are available in **Supplementary Table 2**. Statistical analysis in the vagina of certain subsets was not possible (indicated as N/A in **Supplementary Table 2**), because the frequencies of cells in some animals at some timepoints were below the minimum threshold. (d) Subset frequencies of CD16$^-$ (classical; orange) and CD16$^+$ (intermediate, non-classical; gray)

within total monocytes. Solid (females) and dashed (male) median lines with interquartile ranges (n=4/group).

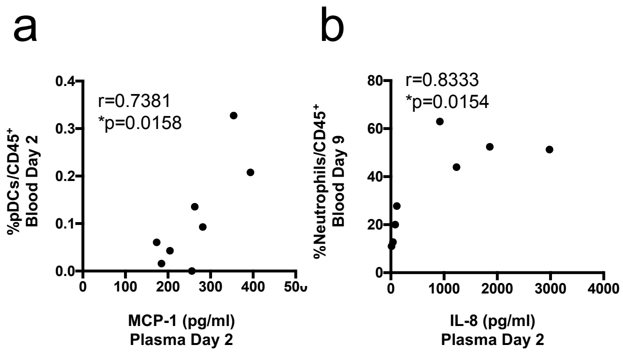

Supplementary Figure 5. Immune parameters in blood correlating with plasma cytokines/chemokines. (a) Correlation between the concentration of plasma MCP-1 measured at 2 dpi and frequencies of pDCs in the blood at 2 dpi. (b) Correlation between the concentration of plasma IL-8 measured at 2 dpi and frequencies of neutrophils in the blood at 9 dpi. Spearman's rank correlation coefficients are shown; unadjusted p-values are shown, both significant (*p≤0.05).

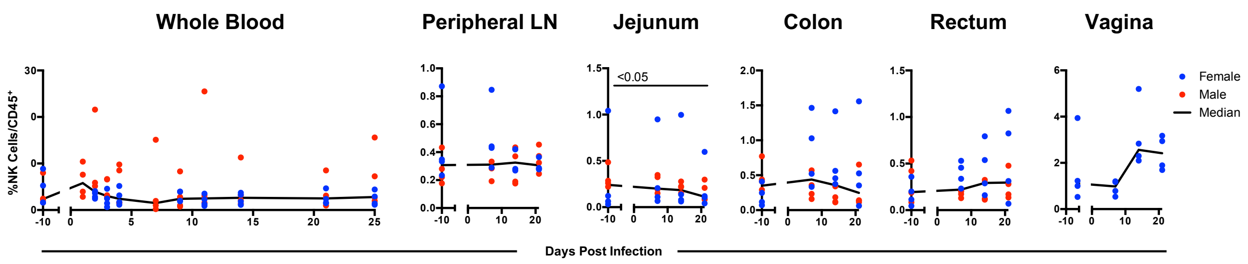

Supplementary Figure 6. NK cells do not significantly increase in response to Zika infection. NK cell frequencies (CD14$^-$CD3$^-$NKG2A$^+$CD8α$^+$) of CD45$^+$ leukocytes over time after ZIKV infection in the whole blood, peripheral LN, and mucosal tissues were evaluated by flow cytometry. Blue (females) and red (males) symbols represent individual animals, with the line indicating the median response over time. Unadjusted p-values ≤0.05 are displayed, and all unadjusted p-values are available in **Supplementary Table 2**.

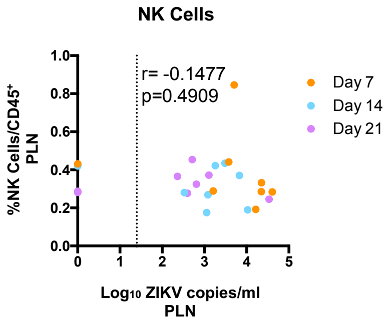

Supplementary Figure 7. Frequencies of NK cells do not correlate with viral burden in the peripheral lymph node. No correlation between viral burden in the PLN (0-21 dpi) and the frequency of NK cells in the PLN between 7-21 dpi. Spearman's rank correlation coefficient and p-value are shown.

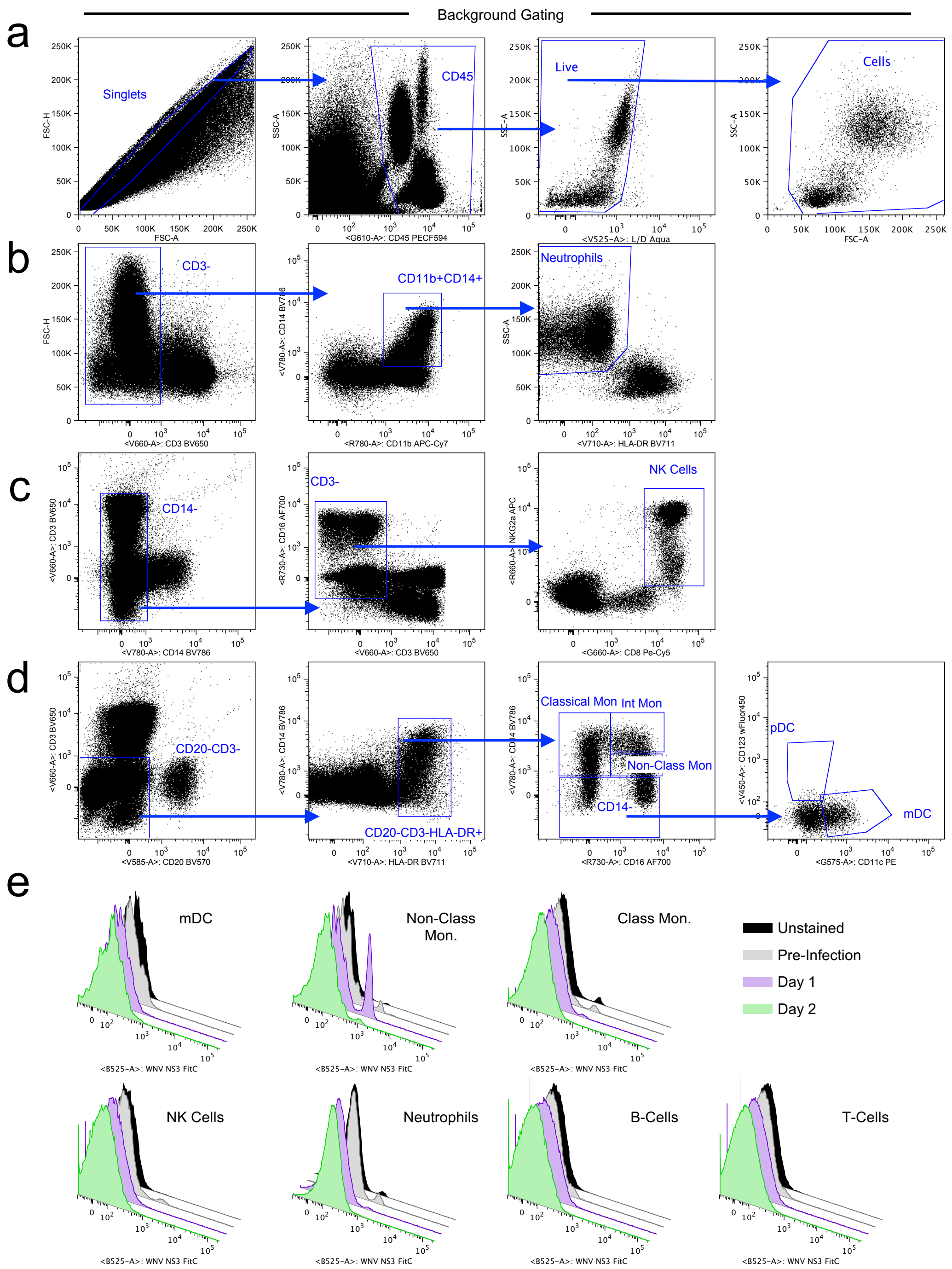

Supplementary Figure 8. Gating Strategies used for immune analyses. Representative gating strategy in blood to identify (a-d) innate immune cells used in Figures 2, 4, and 5 and Supplementary Figures 2-6 and (e) WNV-NS3 positive cells in blood and tissues used in Figure 4. (a) Background gating on FSC singlets, then CD45$^+$ cells, live cells, and cells according FSC-

A and SSC-A profiles were performed on all cell types prior to identification of innate cell subsets. (b) Neutrophils were identified as $CD3^-$, $CD11b^+CD14^+$ and then selected for high SSC-A. (c) NK cells were identified as $CD14^-$, $CD3^-$, and then $NKG2A^+CD8\alpha^+$. (d) Monocytes and DCs were first gating as $CD20^-CD3^-$, $HLA\text{-}DR^+$ and then divided into $CD14^\pm CD16^\pm$ for monocytes and $CD14^-$ for DCs. Monocytes (Mon) subsets were gated into classical ($CD14^+CD16^-$), intermediate (Int) ($CD14^+CD16^+$), and non-classical (Non-Class) subsets ($CD14^{int}CD16^+$) and dendritic cells were gated into pDCs ($CD123^+CD11c^-$) and mDCs ($CD123^-CD11c^+$). (e) $NS3^+$ cells within cellular subsets were identify using histogram analysis. NS3 positive cells after ZIKV infection were identified after background (unstained, black) and pre-infection/baseline subtraction (gray) and meeting a cellular threshold (≥100 cells/gate). Examples of staining for Day 1 (purple) and Day 2 (green) post-ZIKV infection are given. Non-classical monocytes (Non-Class Mon.), classical monocytes (Class Mon.)

Cell Subset	Blood	PLN	Rectum	Jejunum	Colon
pDCs	***0.0286***	0.8857	0.3429	0.2000	***0.0286***
mDCs	0.2000	0.1143	0.3429	0.2000	0.3429
Monocytes	0.6857	0.4857	***0.0286***	***0.0286***	***0.0286***
Classical Mon.	0.4857	0.1143	***0.0286***	***0.0286***	***0.0286***
Intermediate Mon.	0.8857	0.3429	***0.0286***	0.8857	***0.0286***
Non-Classical Mon.	0.4857	***0.0286***	0.0571	***0.0286***	***0.0286***
Neutrophils	***0.0286***	0.3429	***0.0286***	***0.0286***	***0.0286***
NK Cells	0.4857	0.2000	0.4857	0.3429	0.3429

Supplementary Table 1. Comparison of cellular innate responses between males and females in different tissues. Unadjusted Wilcoxon test p-values comparing AUC of cell frequencies of $CD45^+$ leukocytes in blood (-10 to +4 dpi) and tissues (-10 to +21 dpi) between males and females, with $p \leq 0.05$ indicating significant differences (italicized and bolded) and $p \leq 0.08$ indicating trending differences (italicized). Colors indicate the direction of difference, i.e. higher in females (blue) or males (red). Monocytes (Mon.)

Days Post Infection	pDCs	mDCs	Monocytes	Neutrophils	NK Cells	Classical Monocytes	Int. Monocytes	Non-Class. Monocytes
**Whole Blood**								
**1**	0.1484	0.6406	***0.0078***	0.8438	N/A	***0.0078***	0.4609	0.7422
**2**	0.0781	0.5469	***0.0078***	0.7422	0.2500	***0.0078***	***0.0078***	***0.0391***
**3**	***0.0156***	0.8438	***0.0078***	0.7422	0.5469	***0.0078***	0.0547	***0.0078***
**4**	***0.0078***	0.4609	***0.0078***	0.6406	0.6406	***0.0078***	***0.0078***	0.1953
**9**	0.8438	0.0781	***0.0391***	***0.0078***	0.3125	0.1484	***0.0156***	***0.0156***
**11**	0.0781	0.5469	***0.0078***	0.6406	0.7422	***0.0078***	***0.0078***	0.1953
**14**	0.0547	0.2500	0.1094	0.7422	0.6406	0.1094	0.9453	0.9453
**21**	0.1484	0.0781	0.0781	0.5469	0.7422	0.0781	0.1484	0.3125
**25**	0.2500	0.2500	***0.0156***	0.7422	0.8438	***0.0156***	***0.0078***	0.5469
**PLN**								
**7**	0.5469	0.1484	*0.0078*	0.7422	0.5469	***0.0078***	0.2500	0.5469
**14**	0.8438	0.5469	***0.0156***	0.5469	0.9453	***0.0156***	0.9453	0.7422
**21**	0.3125	0.4609	***0.0156***	0.3125	0.6406	0.1250	0.9453	0.3125
**Rectum**								
**7**	***0.0156***	0.3125	0.7422	***0.0156***	0.8438	0.2500	0.7422	0.6406
**14**	0.1094	0.5469	***0.0234***	***0.0156***	0.5469	0.3125	0.0781	0.5469
**21**	0.2500	***0.0078***	0.0781	0.1484	0.3125	0.7422	0.1484	0.5469
**Jejunum**								
**7**	***0.0078***	0.2500	0.4609	***0.0156***	0.5469	***0.0078***	0.8438	0.0781
**14**	***0.0078***	0.8438	0.1484	***0.0078***	0.4609	0.3125	0.6406	0.1484
**21**	***0.0391***	0.8438	***0.0078***	***0.0078***	***0.0391***	0.3125	0.0781	***0.0078***
**Colon**								
**7**	***0.0156***	0.5469	0.0781	***0.0391***	0.3125	0.1094	0.2500	0.8438
**14**	***0.0391***	0.8438	0.8438	***0.0156***	0.7422	0.5469	0.2500	0.7422
**21**	0.1484	***0.0078***	0.1484	***0.0078***	> 0.9999	0.4609	0.5469	0.1484
**Vagina**								
**7**	N/A	N/A	> 0.9999	0.1250	0.2500	> 0.9999	0.2500	0.2500
**14**	N/A	N/A	N/A	0.2500	0.2500	N/A	N/A	N/A
**21**	N/A	N/A	0.2500	0.1250	0.8750	0.2500	0.2500	0.2500

Supplementary Table 2. Comparison of cellular responses between baseline and days post infection across tissues. Unadjusted Wilcoxon test p-values comparing cell frequencies of $CD45^+$ leukocytes in blood, PLN, rectum, jejunum, colon, and vagina between baseline and days post ZIKV infection, with $p \leq 0.05$ indicating significantly different than baseline differences (italicized and bolded) and values indicate trending ($p \leq 0.08$, italicized). Colors indicate the direction of difference i.e. higher (green) or lower (red) after baseline. N/A indicates stats not available due to insufficient numbers of animals at indicated timepoints. Intermediate (Int.), non-classical (Non-Class.)

Days Post Infection	pDCs	mDCs	Classical Monocytes	Int. Monocytes	Non-Class. Monocytes
**CD86 MFI Whole Blood**					
**1**	N/A	***0.0078***	***0.0078***	0.0625	0.1953
**2**	N/A	***0.0078***	***0.0234***	***0.0313***	0.0625
**3**	N/A	0.5469	***0.0156***	***0.0156***	***0.0313***
**4**	N/A	0.6406	0.0547	***0.0156***	0.7188
**9**	N/A	0.3828	***0.0391***	***0.0156***	0.2500
**11**	N/A	0.1484	***0.0078***	***0.0156***	0.4609
**14**	N/A	***0.0391***	***0.0234***	0.1563	0.9375
**21**	N/A	***0.0156***	***0.0234***	***0.0156***	0.8125
**25**	N/A	0.1484	***0.0234***	0.0625	0.5469

Supplementary Table 3. Comparison of CD86 MFI expression between baseline and days post infection in blood. Unadjusted Wilcoxon test p-values comparing CD86 MFI on innate cell subsets in blood, between baseline and days post ZIKV infection, with p≤0.05 indicating significant differences (italicized and bolded) and values indicate trending (p≤0.08, italicized). Colors indicate the direction of difference i.e. lower (red) values after baseline. N/A indicates stats not available due to insufficient numbers of animals at indicated timepoints. Intermediate (Int.), non-classical (Non-Class.)

	pDCs	mDCs	Mon.	Class. Mon.	Int. Mon.	Non-Class. Mon.	Neut.	NK Cells
**p-value**	0.673	0.762	0.077	0.167	0.089	0.077	0.167	0.619
**Spearman r**	-0.191	-0.136	-0.682	-0.546	-0.655	-0.682	-0.546	-0.218

Supplementary Table 4. Correlation between viral load and innate cellular responses in rectum. Spearman's rank correlation coefficient and unadjusted p-value are shown between viral load in rectum at 7 dpi with AUC (-10 to +21 dpi) of cell frequencies of $CD45^{+}$ leukocytes in rectum. Monocytes (Mon.), classical (Class.), intermediate (Int.), non-Classical (Non-Class.), neutrophils (Neut.).

Assay	Primer Name	Sequence 5’ – 3’
prME	ZIKV 1087 FWD (10 μM)	CCGCTGCCCAACACAAG
	ZIKV 1163c REV (10 μM)	CCACTAACGTTCTTTTGCAGACAT
	ZIKV 1108 FAM/no quencher	AGCCTACCTTGACAAGCAGTCAGACACTCAA

Supplementary Table 5. ZIKV primers used in study.

Antibody	Clone	Amount Used (µL)/ Dilution	Catalog #	Company
NKG2A	Z199	10	A60797	Beckman Coulter
CD16	3G8	4	302026	BioLegend
CD20	2H7	2.5	302332	
CD4	OKT4	2.5	317438	
HLA-DR	L243	2.5	307644	
CD14	M5E2	2.5	301840	
Streptavidin	-	1:200	405201	
CD11b	ICRF44 (44)	5	557754	BD BioSciences
CD3	Sp34-2	2.5	563916	
CD11c	S-HCL-3	20	347637	
CD45	D058-1283	2.5	562394	
CD123	6H6	2.5	48-1239-42	eBioscience
CD8α	RPA-T8	5	15-0088-42	
CD86	IT2.2	5	46-0869-42	
Anti-WNV NS3	Polyclonal	1:100	BAF2907	R&D Systems

Supplementary Table 6. Antibodies used in study.

Supplementary Methods.

Enzyme-linked immunosorbent assay

Plasma IgG and IgA levels against the Zika virus E protein were quantified by an Enzyme-Linked Immunosorbent Assay (ELISA). NHP plasma was added to a 96-well plate (Costar) pre-coated with recombinant 50 ng Zika E protein (Fitzgerald). IgG and IgA specific for Zika E protein were detected by a Horseradish peroxidase-linked antibody (IgG: ThermoFisher, IgA: Rockland-inc.) via a color change reaction upon addition of the SureBlue Reserve substrate (KPL). Reaction was stopped with 1 N HCl (VWR) and absorbance at 450 nm was measured on an EMax plate reader (Molecular Devices) and compared to an IgG (MyBioSource) or IgA (MassBiologics) standard curve to calculate Ig levels.
